# Transcatheter Closure of PFO and ASD: Multimodality Imaging for Patient Selection and Perioperative Guidance

**DOI:** 10.3390/jcdd8070078

**Published:** 2021-07-03

**Authors:** Gabriele Egidy Assenza, Luca Spinardi, Elisabetta Mariucci, Anna Balducci, Luca Ragni, Cristina Ciuca, Roberto Formigari, Emanuela Angeli, Gianfranco Vornetti, Gaetano Domenico Gargiulo, Andrea Donti

**Affiliations:** 1 Pediatric Cardiology, Pediatric Cardiac Surgery and Adult Congenital Heart Disease Program, Department of Cardio—Thoracic and Vascular Medicine, IRCCS Azienda Ospedaliero—Universitaria di Bologna, Via G. Massarenti, 9, 40138 Bologna, Italy; elisabetta.mariucci@aosp.bo.it (E.M.); anna.balducci@aosp.bo.it (A.B.); luca.ragni@aosp.bo.it (L.R.); cristina.ciuca@aosp.bo.it (C.C.); emanuela.angeli@aosp.bo.it (E.A.); gaetano.gargiulo@aosp.bo.it (G.D.G.); andrea.donti@aosp.bo.it (A.D.); 2 Neuroradiology Program, IRCCS Azienda Ospedaliero—Universitaria di Bologna, 40138 Bologna, Italy; luca.spinardi@aosp.bo.it (L.S.); gianfranco.vornetti@gmail.com (G.V.); 3 Interventional Cardiology, Cardiology and Cardiac Surgery Program, Ospedale Bambino Gesu’, 00165 Roma, Italy; roberto.formigari@opbg.net

**Keywords:** patent foramen ovale, atrial septal defect, multimodality imaging, transcatheter closure

## Abstract

Transcatheter closure of patent foramen ovale (PFO) and secundum type atrial septal defect (ASD) are common transcatheter procedures. Although they share many technical details, these procedures are targeting two different clinical indications. PFO closure is usually considered to prevent recurrent embolic stroke/systemic arterial embolization, ASD closure is indicated in patients with large left-to-right shunt, right ventricular volume overload, and normal pulmonary vascular resistance. Multimodality imaging plays a key role for patient selection, periprocedural monitoring, and follow-up surveillance. In addition to routine cardiovascular examinations, advanced neuroimaging studies, transcranial-Doppler, and interventional transesophageal echocardiography/intracardiac echocardiography are now increasingly used to deliver safely and effectively such procedures. Long-standing collaboration between interventional cardiologist, neuroradiologist, and cardiac imager is essential and it requires a standardized approach to image acquisition and interpretation. Periprocedural monitoring should be performed by experienced operators with deep understanding of technical details of transcatheter intervention. This review summarizes the specific role of different imaging modalities for PFO and ASD transcatheter closure, describing important pre-procedural and intra-procedural details and providing examples of procedural pitfall and complications.

## 1. Introduction

Transcatheter closure of patent foramen ovale (PFO) and secundum type atrial septal defect (ASD) are nowadays performed in many centers throughout the world even in non-pediatric/congenital interventional laboratories. ASD closure is justified in the presence of significant left-to-right shunt across the atrial communication with evidence of right ventricular volume overload and normal pulmonary vascular resistance (Figure S1 Online Supplementary Material) [1]. PFO closure has been shown to reduce recurrent cerebrovascular/systemic event in young patients presenting right-to-left shunt across PFO tunnel with recent history of embolic stroke/systemic arterial embolization, high probability for paradoxical embolization event (absence of alternative embolic sources such as atrial fibrillation, aortic plaques, extracranial carotid atherosclerotic disease, cardiac tumor, valvular pathology, pulmonary artero-venous fistulas) and no significant vascular pathology (carotid or vertebral artery dissection) (Figure S1 Online Supplementary Material) [2]. Marginal indications for PFO closure include platypnea orthodeoxia and unexplained acute decompression illness in professional scuba divers [2].

Although ASD and PFO echocardiographic imaging has been extensively discussed and reported, proper patient selection, procedural guidance, and follow-up monitoring are strongly influenced by different cardiac and non-cardiac imaging modalities that include transthoracic (TTE)/transesophageal (TEE)/intracardiac echocardiography (ICE), vascular Doppler studies, neuroimaging studies, transcranial Doppler study (TCD) (Figure S1 Online Supplementary Material).

Familiarity with these imaging techniques is of pivotal importance for practioners and interventionalists involved in this field. This article reviews clinical indication to appropriate imaging studies, proper interpretation of imaging findings, and it exemplifies possible pitfalls in imaging interpretation along with possible procedural complications.

## 2. Focused Anatomy of Atrial Septum and ASD

**Figure 1:**
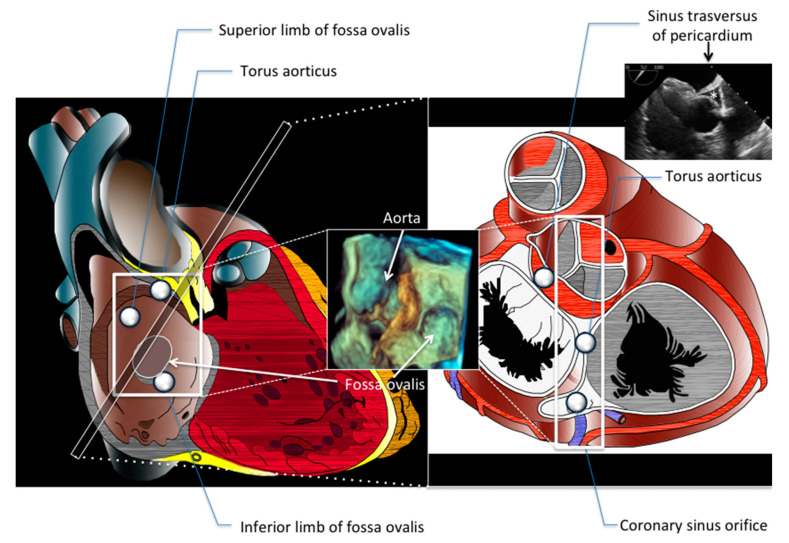
Anatomy of atrial septum. Left panel shows right atrial view of atrial septum and anatomic relationship with superior vena cava, inferior vena cava, coronary sinus. The bulging of aorta toward the atrial septum (torus aorticus) is shown. The central insert is a TEE three-dimensional view showing the relationship between aortic root and fossa ovalis. The right panel represents a cross-anatomical section set at the level shown in the left panel. The extracardiac space between the aortic root (anteriorly) and atrial chambers (posteriorly) is the sinus transversus of pericardium. A TEE view of such space is presented in the right-top insert (*).

**Figure 2:**
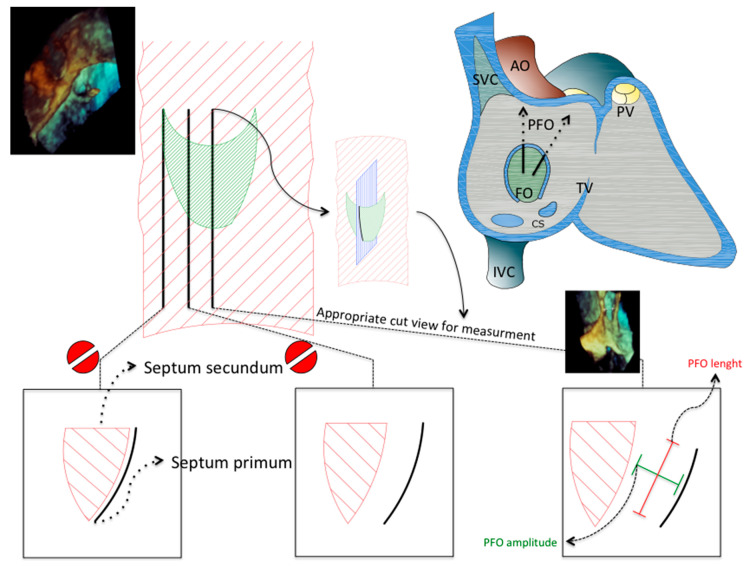
Anatomy of PFO. Detailed anatomy of PFO tunnel is presented. Three different cross-section planes are shown located at three different levels of septum primum. Due to the particular anatomy of septal attachment, central plane should be used for procedural measurement of PFO amplitude and length for proper device selection. Selected three-dimensional TEE left atrial view of PFO and appropriate cut for PFO amplitude measurement are presented. AO = AOrta; CS = Coronary Sinus; FO = Fossa Ovalis; IVC = Inferior Vena Cava; PFO = Patent Foramen Ovale; PV = Pulmonary Valve; SVC = Superior Vena Cava; TEE = Trans-Esophageal Echocardiography; TV = tricuspid valve.

Anatomy of atrial septum and PFO is presented in Figure 1 and Figure 2 and Video S1 Online Supplementary Material [2,3,4]. Please see Supplementary Material for additional details.

**Figure 3:**
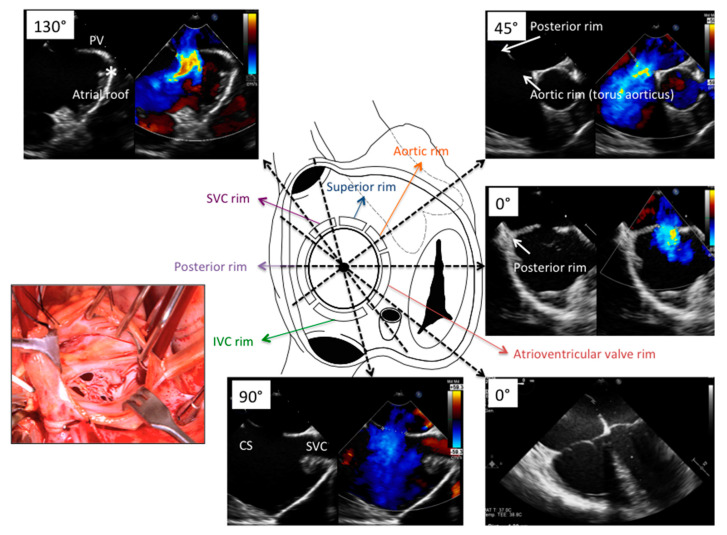
Atrial septal defect and surrounding borders. Right atrial view of atrial septum is shown along with relationship between atrial septal defect and surrounding borders. Appropriate and border-specific TEE views are presented. Anatomical view is provided for comparison (left insert). TEE = Trans-Esophageal Echocardiography.

Secundum type ASD is related to embryonic deficiency of septum primum, accordingly the defect is usually located or at least involving the central portion of atrial septum (Figure 3). Wide anatomic variation is present in relation to the size, location, quality of surrounding borders, and distance between defect and important cardiac structures such as torus aorticus, roof of atrial septum, coronary sinus, superior vena cava, and atrio-ventricular valves (Figure 3). These anatomical details are of paramount importance for interventionalist and imager and they guide patient selection, procedure monitoring, and propensity to procedural complication (such as device embolization, device erosion, residual shunt).

Standard views for transesophageal echocardiographic evaluation of atrial septum are reported in Table 1 [5].

## 3. PFO

### 3.1. Indication: Patient Screening and Standardized Approach to PFO-Related Event

PFO transcatheter closure should be reserved to young patients with a diagnosis of embolic stroke/event of unknown source (ESUS/systemic embolization), tailoring down those with a high probability for a PFO-related embolic event, where PFO closure proved to be superior to medical therapy in reducing the risk of recurrence [2,6]. The complex diagnostic work-up for such patients is beyond the scope of this article and it has been extensively discussed in other documents [2].

However, ischemic stroke can result from a variety of causes, such as large artery atherosclerosis, small vessel occlusion, and cardioembolism. Although no truly specific neuroradiological pattern of PFO-related stroke has been demonstrated, reviewing neuroimaging studies is pivotal to correctly identify potentially treatable patients [3]. The main goal of neuroimaging in the evaluation of patients considered for PFO closure is confirming the presence of an ischemic lesion as well as ruling out non-embolic causes of stroke.

**Figure 4:**
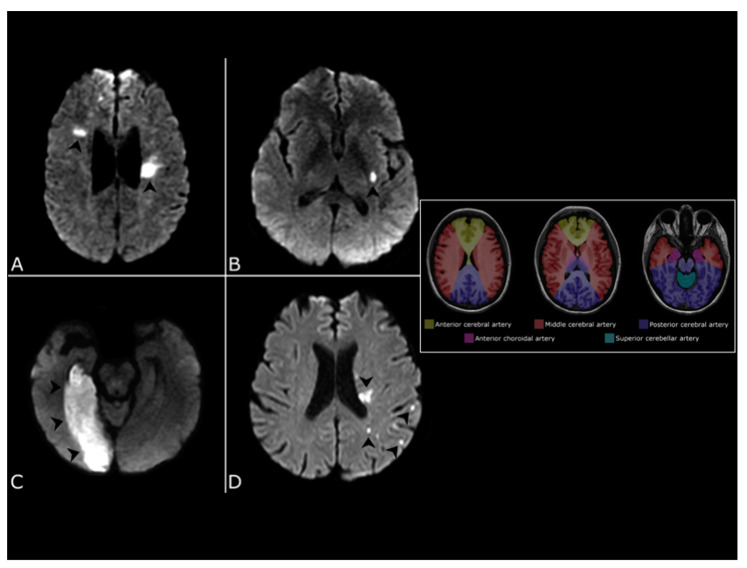
Neuroimaging features of embolic lesion. Diffusion-weighted MRI showing different lesion (arrowheads) patterns. (**A**) bilateral lesions in the right and left middle cerebral artery territory. (**B**) small subcortical lesion. (**C**) large corticosubcortical lesion in the right posterior cerebral artery territory. (**D**) multiple small lesions in the left middle cerebral artery territory. A cartoon showing cerebral vascular territories at the level of the body of the lateral ventricles (**left image**), at the level of the basal ganglia and internal capsule (**center image**) and at the level of the mesencephalon (**right image**) is provided.

An ischemic lesion is defined as superficial when it involves the cerebral or cerebellar cortex. Other locations, including the noncortical gray matter (thalami and basal ganglia) and deep white matter in the cerebrum or cerebellum are considered deep (Figure 4).

Diffusion-weighted magnetic resonance imaging (DWI) is superior to computed tomography for the detection of ischemic lesions in the first hours after the onset of clinical symptoms as well as in differentiating between chronic and acute lesions (Figure 4) [7]. Furthermore, DWI is superior in the identification of very small clinically silent lesions, which may influence the diagnosis of stroke subtype [8].

In the vast majority of cardioembolic strokes, one or more cortical or corticosubcortical lesions are present. The identification of multiple acute infarctions involving both right and left anterior or both anterior and posterior circulations is highly suggestive for a cardiac source of emboli (Figure 4). Conversely, multiple lesions involving a single vascular territory are typical of artery-to-artery embolism in the setting of large artery atherosclerosis [9].

When a single corticosubcortical lesion is present, potential etiologies include both cardiac pathology and large artery atherosclerosis. In these instances, head and neck arteries imaging is needed to assess for the presence of atherosclerotic plaque in a vessel supplying the infarcted area [10].

Furthermore, in the posterior circulation cardioembolic lesions show a preferential involvement of the superior cerebellar artery territory compared to other etiologies [9].

Lesion size may offer further clues, since emboli from a cardiac source have been found to result in larger corticosubcortical lesions in respect to those secondary to atherothrombosis.

The finding of small deep infarcts, called lacunar strokes (and less than 15–20mm in size) is a challenging quest for differential diagnosis [11]. These are small ischemic lesions involving the deep gray matter or the subcortical white matter caused by the occlusion of a single perforating artery usually from microatheroma or lipohyalinosis. This etiology is also supported by the identification of other imaging features of cerebral small vessel disease, such as white matter hyperintensities and prominent perivascular spaces [12].

Patients with PFO-related stroke show a higher frequency of ischemic lesions > 15mm, with cortical involvement and without coexisting older ischemic lesions [3].

Among the cardioembolic stroke patterns, PFO-related events are more often limited to the cortex or may present scattered pattern with multiple and small (<15 mm) lesions, while atrial-fibrillation related lesions are usually larger with corticosubcortical distribution [13]. Additionally, PFO-related strokes occurred more frequently in the vertebrobasilar circulation, a finding compatible with the reported increased blood flow to this vascular territory after the Valsalva maneuver.

Furthermore, a higher frequency of multiple cortical lesions in patients with PFO-related stroke was associated with greater amount of right-to-left shunt as well as the presence of atrial septum aneurysm.

**Figure 5:**
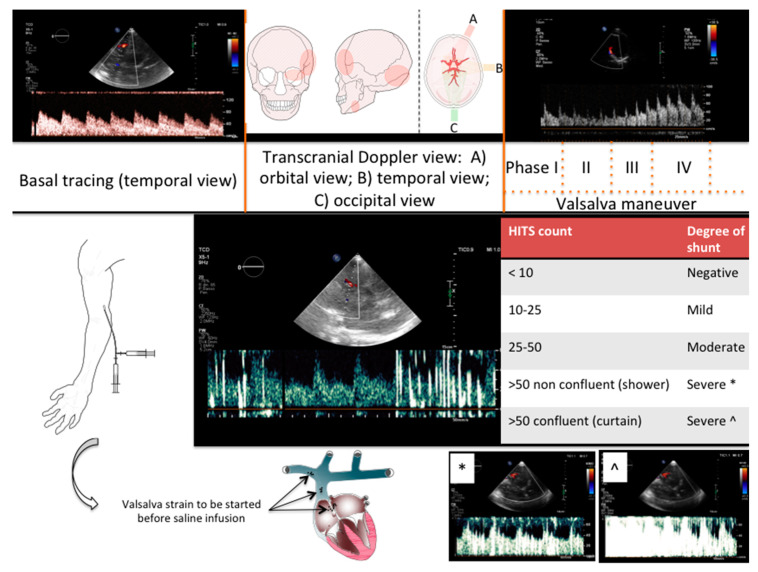
Transcranial Doppler Study. Top row (left to right) shows basal TCD tracing, routine transcranial acoustic window and Valsava maneuver effect on TCD tracing. Bottom row shows suggest site for peripheral vein cannulation, mechanism of Valsalva-mediated effect on PFO-associated paradoxical embolization. The central tracing depicts a moderate shunt. Suggested threshold for TCD shunt grading with pertinent samples tracings are also presented. HITS: High Intensity Transient Signals; TCD = Trans-Cranial Doppler.

A key decision element for proper PFO closure indication is to provide evidence of important right-to-left shunt occurring at the level of PFO tunnel [2]. TCD proved to be a sensitive method to detect right-to-left shunt, although it does not provide exact location of shunt occurrence (Table 2). We usually perform TCD in an ambulatory setting, using transtemporal (middle cerebral artery) or transorbital (oftalmic artery) window (Figure 5). After large vein peripheral cannulation (using a 18 Gauge peripheral cannula for high flow injection), an agitated solution of saline, blood, and air is injected as contrast media in basal condition and after Valsalva maneuver (Figure 5, Video S2 of the Online Supplementary Material). In selected cases with sub-optimal transtemporal or transorbital windows, internal carotid Doppler may be used as alternative. Meticulous care should be placed in obtaining a foam rich solution (with high resonance properties) and setting the appropriate equipment parameter including a low signal amplification to enhance detection of high intensity signal super-imposed to the baseline Doppler tracing. Emphasis needs to be placed on appropriate patient coaching for the Valsalva maneuver (Figure 5 and Video S2 Online Supplementary Material). A correctly performed strain maneuver should always have clear reflection on Doppler tracings with clear recognition of phase III and IV (overshooting) (Figure 5). Ideally the assistant should inject the media immediately after phase II leading to the accumulation of the saline solution within the superior vena cava. After strain release (which is usually timed by the operator), the column of contrast will travel to the right atrium during the opening of the PFO tunnel simulating the physiologic behavior of paradoxical embolization and increasing shunt detection if present (Figure 5 and Video S3 Online Supplementary Material) [14] The presence of large shunt during basal infusion (without Valsalva) must always raise suspicion of unusual shunt location such as pulmonary artero-venous fistulas, because this may have important impact on patient care and procedural planning (Video S4 Online Supplementary Material). It is reasonable to complete the examination performing a saline injection (both at basal condition and during Valsalva) during transthoracic 4-chamber view imaging, to confirm the intracardiac shunt location. It is reasonable to consider PFO closure only in patients with significant TCD detected shunt (Figure 5).

TEE is required before final decision to PFO closure (Table 2). It is reasonable to consider TEE after a positive TCD (unless TEE is indicated for other reasons) to avoid un-necessary TEE in patients without evidence of significant shunt. Pre-closure TEE has three main purposes: (1) to confirm the presence of PFO, allowing for complete anatomical delineation of the tunnel and surrounding structure (length and amplitude of the tunnel, accessory fenestration, septum primum hypermobility or aneurysms, Chiari network, large Eustachian valve); (2) to exclude any other intracardiac source of systemic embolization including left atrial appendage/atrial thrombosis, mitral or aortic valve pathology, cardiac tumor (atrial mixoma, aortic valve fibroelastomas); (3) to exclude complex atherosclerotic pathology of the aortic arch (Figure S2 Online Supplementary Material). Intravenous bubble contrast administration may be repeated at the time of pre-closure TEE, in particular if there is unclear source/degree of shunt. We generally rely less on TEE for shunt magnitude evaluation because of many possible confoundings (single plane examination, patient sedation with difficulties in performing a good Valsalva, more complex coordination between Valsalva and contrast injection, image clouding during Valsalva).

A subgroup of patient is referred to PFO closure for platypnea orthodeoxia [15,16]. These patients usually present with very large PFO tunnel, enlarged aortic root, advanced age and some precipitating factors such as abdominal/thoracic surgery, prolonged hospitalization, diaphragmatic paralysis, hepatomegalia, scoliosis [15]. PFO closure in this setting present higher technical complexity, but the clinical outcome is generally most rewarding with a substantial improvement in hypoxemia and some residual shunt (which is somewhat common) is usually well tolerated [15].

### 3.2. Perioperative Monitoring

Perioperative monitoring is usually accomplished via TEE or ICE [17] ICE has the advantage of avoiding general anesthesia and it may expedite the procedure. Main goals of such monitoring are: (1) confirm pre-closure findings; (2) assist interventionalist during transcatether deployment of closing device; (3) confirm proper device positioning, rule-out substantial residual shunt, and exclude abnormal interaction between device and surrounding structure. Table 3 summarizes an intraprocedural checklist that should usually be accomplished before the interventionalist takes vascular access. Full scout of atrial septum, starting at 0° (low/middle/high esophageal views), will be followed by careful view change with planar rotation (using 15° incremental step) to allow for proper multiplanar reconstruction of atrial septum. After clearance from imager, vascular access is taken usually with ultrasound guidance and a repeated contrast study is performed flushing the contrast media from the side port of the vascular sheath placed in the femoral vein.

**Figure 6:**
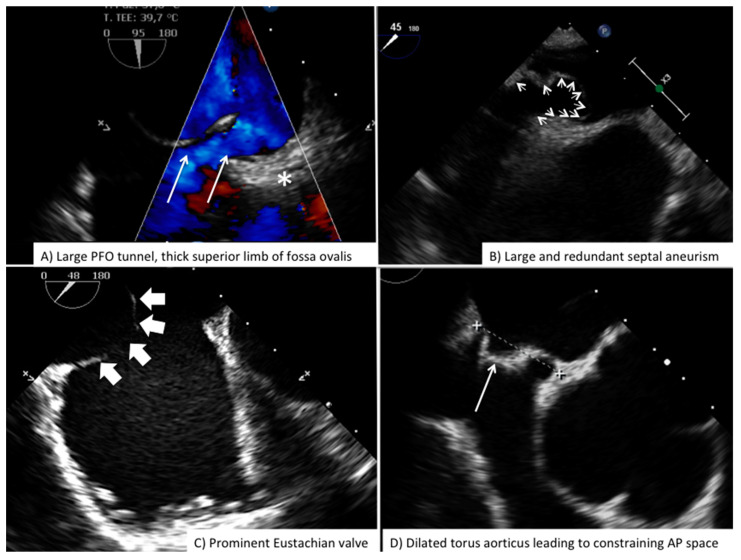
Challenging anatomies for PFO closure. Common anatomical features increasing procedural complexity and complication during PFO closure. PFO = Patent Foramen Ovale.

Procedural monitoring is usually carried using three main TEE views: (1) mid-esophageal long axis (90° bicaval view) of the atrial septum; (2) mid-esophageal short axis (45°–60° aortic view) of the atrial septum; (3) mid-esophageal 0° that is particularly useful to scan the posterior border of atrial septum (Table 2). Imager must confirm proper engagement of PFO tunnel by the wire. This is a key detail because, in the presence of accessory fenestration, wrong wire positioning within the fenestration will leave the PFO tunnel not engaged by the device and this will increase risk of device embolization and large residual shunt after deployment (Figure S3 Online Supplementary Material). Correct wire positioning across PFO tunnel is usually associated with enhanced separation between septum primum and superior limb of the fossa ovalis. The importance of a skilled echocardiographer, accustomed to every single step of the procedure as well as to the device currently in use, cannot be overemphasized. Knowing the differences in the echocardiographic appearance of the several available devices helps to readily recognize potential problems and to anticipate impending complications (Figure S4 Online Supplementary Material). Periprocedural imaging provides important information for the interventionalist regarding proper device to be used in that particular patient: (1) thickness of superior limb of the fossa ovalis (septum secundum); (2) length and amplitude of PFO tunnel during wire sizing; (3) hypermobility of septum primum or presence of septal aneurysm; (4) degree of shunt; (5) distance between the torus aorticus and posterior wall of atria (Figure 6). Fluoroscopic monitoring is essential, PFO crossing and subsequent device delivering is usually accomplished using left oblique anterior view with some cranial angulation. During deployment the imager needs to maintain the tip of the guiding sheath at the center of the image so to allow the interventionalist to avoid any direct contact between device and atrial wall. After deployment of left atrial disc, direct echocardiographic guidance will allow the interventionalist to retract the device-guiding sheath as a unit so to allow for complete contact of left atrial disk with atrial septum, often proper clockwise or counterclockwise rotation of the device-guiding sheath unit may be necessary to optimize proper alignment. After release of the right atrial disk, complete reassessment of device grasp onto the septum is of paramount importance and will need both bidimensional and color-Doppler scan both in long and short axis views with cranio-caudal swipe. Particular attention should be placed to exclude potential catching of Chiari network or large Eustachian valve within the device, good grasp on the superior and posterior border of atrial septum, complete coverage of the entire tunnel length, relatively well formed and parallel disks and absence of interference with surrounding cardiac structure (superior and inferior vena cava, coronary sinus, atrio-ventricular valves).

If the interventionalists chose to use a Gore© CardioForm (W. L. Gore & Associates, Inc. Flagstaff, AZ, USA), a post-deployment contrast study may be performed having the device still attached to the securing lines. We strongly suggest performing such a study, because the presence of large shunt may be associated with undetected device malposition and increased risk of post-closure recurrent event. We perform a saline injection from the large femoral sheath. After complete device release, a final contrast study should be performed along with reassessment of device position and spatial interference with cardiac structure. Figure S5 Online Supplementary Material shows common pitfall during PFO closure including acute device dislocation, residual shunt, and wrong wiring of accessory fenestration.

Final release is usually associated to subtle device repositioning (Video S5 Online Supplementary Material). Accordingly, before closing the case, we usually obtain a 4-chamber transthoracic echocardiographic view of the device that may be very useful in case question of late device malposition/embolization should arise.

## 4. ASD

### 4.1. Indication

**Figure 7:**
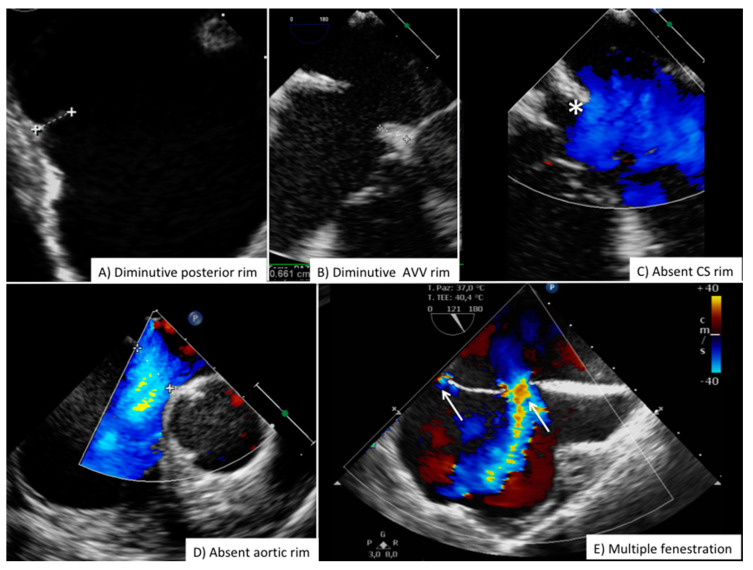
Challenging anatomies for ASD closure. Common anatomical features increasing procedural complexity and complication rate during ASD closure. ASD = secundum type Atrial Septal Defect; AVV = Atrio-Ventricular Valve; CS = Coronary Sinus.

Although there are slight differences between children and adult patients, current indication for ASD closure requires large left-to-right shunt with evidence of right ventricular volume overload and normal pulmonary vascular resistance (Figure S1 Online Supplementary Material) [1,18,19]. Full right cardiac catheterization is usually not required in children and young adults, it may be necessary in older adults or in patients with specific risk factors for pulmonary vascular disease or restrictive left ventricular/atrial physiology [20]. The great majority of patients referred for ASD closure are evaluated with transthoracic echocardiography (Table 2). Pre-closure transesophageal echocardiography is often considered in adult patients with sub-optimal transthoracic windows or in cases with marginal anatomical suitability (Table 2). Figure 7 summarizes pre-ASD frequent high risk or suboptimal anatomical features (we do not recommend routine ASD closure in children less than 15 Kg of weight, and in children <30 Kg some restriction may apply depending on the relative ASD size compared to body surface area and surrounding structure) [21]. Pre-procedural measurement of the length of atrial septum in 4-chamber transthoracic view will help to ensure that the hypothetical left atrial disc (which can be up to 16mm larger than device waist) will fit.

We recommend to routinely check for sign of anomalous pulmonary venous return, that should be suspected if there is discrepancy between ASD size and degree of right volume overload, or in case of enlargement/abnormal flow in the innominate vein, superior vena cava, inferior vena cava or coronary sinus. Similarly sinus venosus defect must be always ruled out and in case of concern, cardiac magnetic resonance imaging should be performed to reassess pulmonary vein and defect anatomy (Figure S6 Online Supplementary Material) [19].

### 4.2. Perioperative Monitoring

As discussed for PFO closure, the echocaradiographer needs to be perfectly aware of the technical aspects of the procedure and the planned device (Figure S7 Online Supplementary Material). If ICE monitoring is chosen, the imager needs to be aware that there will be increasing ability to delineate posterior and inferior border of the defect, but complex anatomy with redundant and highly mobile septum may be more challenging during device assessment and intervention.

Periprocedural monitoring must confirm pre-closure findings (always reassess pulmonary vein anatomy before starting the procedure), guide device deployment and confirm absence of residual shunt and abnormal interference between closing device and surrounding structure (Table 2). Three-dimensional (3D)-TEE is now routinely used for assessing complex cardiac anatomy and it may be more useful in complex ASD with very elliptical shape, hypermobile or aneurysmal septum primum or in case of multifenestrated atrial septal defect (Figure S8 Online Supplementary Material) [22]. In case of multifenestrated defect, the imager must confirm with interventionalist which defect is targeted to allow for complete covering of the other defects (usually the central one is chosen and a non self-centering device is used).

We routinely use a combination of bidimensional imaging, color-Doppler imaging, and balloon sizing to guide device choice (Figure S9 Online Supplementary Material). The largest diameter is usually considered. Close attention must be placed in order to avoid balloon overdistension of atrial septal defect by stopping balloon inflation as soon as the shunt disappears on color-Doppler (or even accepting a small amount of persistent left to right shunt in case of a very compliant septum). Moreover, to ensure to be working in a “stop flow” diameter, gentle reduction in balloon pressure should be used to demonstrate early (re)appearance of flow between sizing balloon and defect border. Device oversizing has been associated with an increased risk of atrial wall erosion and should be generally avoided. However, the precise mechanism of wall erosion is still a matter of debate, and the geometric interplay between the device and the aortic wall may even suggest, in very selected cases, a prudent oversizing allowing the aortic root to be embraced by the device, especially in cases of diminutive securing rims occurring in adult patients or children with larger body surface area and permissive anatomy [23].

Routine high risk anatomies include diminutive posterior and SVC rim (<5 mm), very large defect (>30 mm), thin and flimsy rim tissue; the absence of aortic rim is not considered an absolute contraindication but it has been associated to increased risk of erosion and embolization in particular if SVC border is also diminutive or absent (Figure 7) [23].

**Figure 8:**
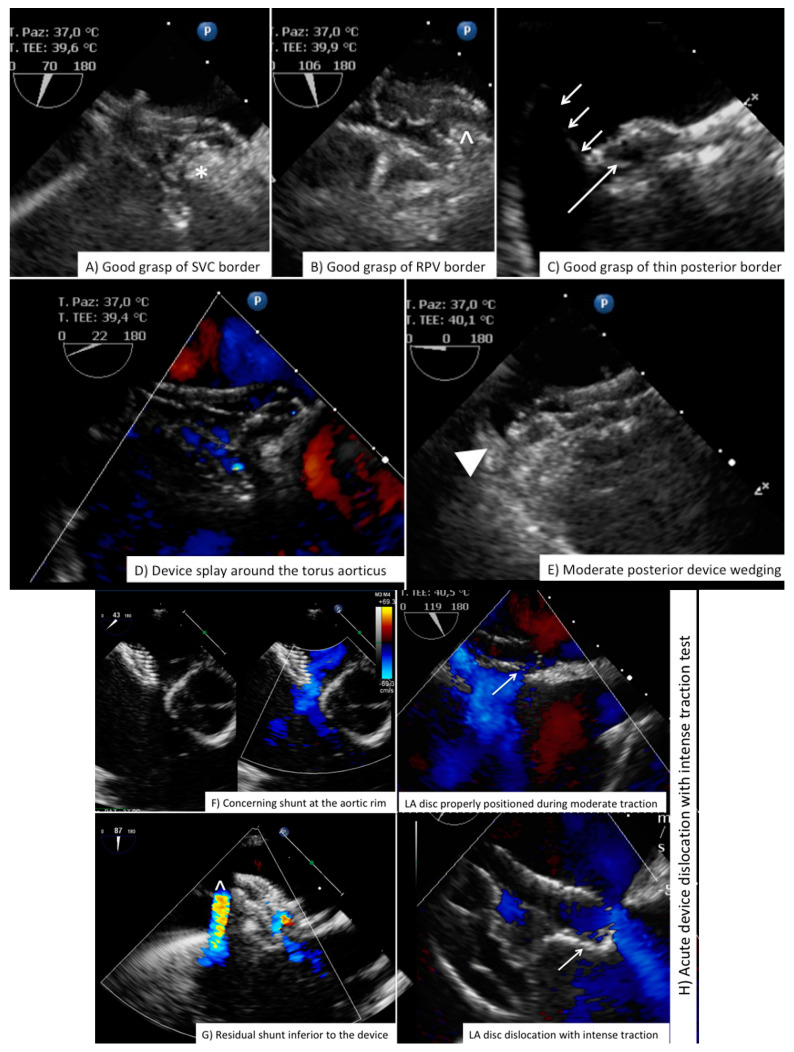

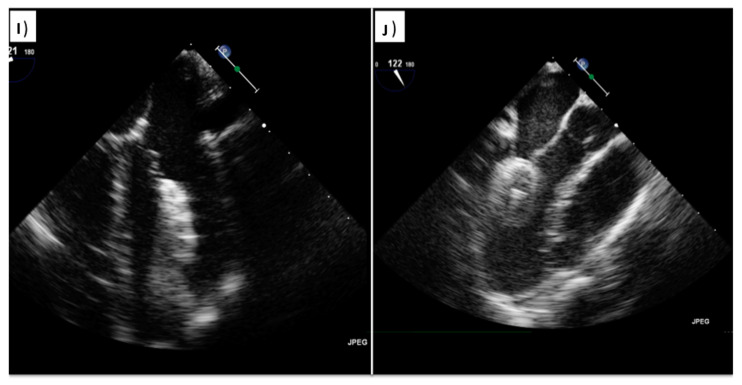
Complication after ASD closure. Examples of complication or sub-optimal procedural result after ASD closure. Please note in (**F**), the presence of shunt between the device and torus aorticus is to be compared with normal “intra-device” flow seen in (**G**). Device embolization to the left ventricular cavity is shown in (**I**,**J**). ASD = secundum type Atrial Septal Defect; LA = Left Atrium; RPV = Right Pulmonary Valve; SVC = Superior Vena Cava.

As described for PFO closure, intraprocedural imaging needs to tailor details that help the interventionalist during deployment and device release (Table 2). Again fluoroscopic monitoring is important, left anterior oblique view is often used with cranial angulation. If balloon sizing is chosen, in order to better delineate balloon waist on the fluoroscopi monitor, caudal angulation may be necessary. In the absence of retroaortic rim, to facilitate good alignment of the device and to optimize grasp to the torus aorticus, some devices can be opened within the right superior pulmonary vein. During this maneuver, the interventionalist will clockwise rotate the guiding sheath so that the tip of the sheath will be seen in the right upper pulmonary vein using the mid-esophageal long axis (90–110° bicaval view) with probe rotation posterior to the superior vena cava. It is important that the imager will follow this clockwise rotation so to keep the tip of the sheath always in the center of image checking that the interventionalist is not opening and pushing the device against the atrial wall. After device deployment, but before device release, a full assessment with bidimensional and color-Doppler is mandatory (Figure 8). Long-axis TEE 140° view may be useful in particular to evaluate device grasp of the pulmonary vein border and rule out abnormal interaction with LA roof (Figure S10 Online Supplementary Material).

Although, some intradevice shunt is frequently seen and it is often due to tension from the delivery cable or securing lines, there are some subtle echo markers of suboptimal position that should be always identified: (1) incomplete grasp on the superior or postero-inferior border; (2) residual shunt viewed at 45°–60° between the device and torus aorticus at baseline and under gentle device traction; (3) shifting device position during pull and push maneuver; (4) interference with mitral valve/superior vena cava/inferior vena cava/coronary sinus; (5) severe device wedging (device not moving throughout the cardiac cycle but entangled between the anterior/aortic and the posterior rim) with direct contact between device and surrounding atrial wall or structures (Figure 8, Videos S6–S8 Online Supplementary Material). In all these cases, an open discussion with interventionalist must occur and potential device repositioning or change may be necessary. After device release an additional complete device evaluation is important because quite often there is some shifting in device positioning after cable or secure lines release, that is usually allowing the device to fit in a more “anatomical” position. Again, we reinforce the importance of final transthoracic 4-chamber view imaging to have a comparison frame if any question of late malposition should arise.

## 5. Training and Quality Measures

Training for proper acquisition and interpretation of each imaging modality has been presented in multiple documents. The specific setting of periprocedural multimodality imaging requires a dedicated collaboration between interventionalists, cardiac imagers, and neuro-radiologists. Although data regarding training in this specific setting are lacking, it may anticipated that a minimum of 10 supervised TCD, 50 supervised pre-closure TEE, and 30 supervised periprocedural TEE are required to become independent operator.

A number of quality measures parameters have been discussed both for imaging and procedural performance [24]. We believe that pre-closure imaging should be able to keep the procedural failure at less than 5% of cases, device embolization should be kept lower than 0.5%, and device erosion should complicate less than 0.1–0.2% of such procedures.

## 6. Final Consideration

Transcatetheter closures of ASD and PFO are established cardiovascular interventions that are now being performed often by adult interventional cardiologist with limited experience to congenital and structural heart intervention. Multimodality imaging is required for proper patient screening, procedure indication and to deliver these transcatheter therapeutics safely and effective. Although dedicated and formal training in high volume centers by expert operators is of primary importance to master these imaging techniques, we believe this document may be useful to review current practice, identify potential gaps in knowledge, and promote clinical improvement in the future.

## Figures and Tables

**Table 1:** Proposed TEE view for ASD/PFO assessment (modified from Silvestry et al. J Am Soc Echocardiogr, 2015) [5].

View	Atrial Septal Anatomy	Procedural Assessment	Suggested Multiplane Angles	Esophageal Position
Basal transverse	SVC, superior aortic, RUPV	Device relationship in atrial roof	0°, 15°, 30°, 45°	Mid- to upper esophagus
Four-chamber	Posterior and AVV rims, maximal ASD diameter	Device relationship to AV valves	0°, 15°, 30°,	Mid-esophagus
Short-axis	Posterior and aortic rims, maximal ASD diameter, PFO tunnel and atrial anterior-posterior distance	Device relationship to AoV and posterior atrial wall	30°, 45°, 60°, 75°	Mid- to upper esophagus
Bicaval	IVC and SVC rims, maximal ASD diameter, PFO amplitude and lenght	Device relationship to RA roof/dome	90°, 105°, 120°	Mid-to upper esophagus and deep transgastric
Long-axis	Dome/roof of LA	Device relationship to LA dome/roof	120°, 135°, 150°	Mid- to upper esophagus

ASD = Atrial Septal Defect; AoV = Aortic Valve; AVV = Atrio-Ventricular Valve; IVC = Inferior Vena Cava; LA = Left Atrium; PFO = Patent Forame Ovale; RA = Right Atrium; RUPV = Right Upper Pulmonary Vein; SVC = Superior Vena Cava.

**Table 2:** Comparison of different imaging modalities for PFO and ASD transcatheter closure.

Procedure	Imaging Modality	Outpatient Clinic Setting	Pre-Procedural Evaluation	Intra-Procedural Evaluation	Quantitative Data	Complexity of Training	Conscious Sedation, Anesthesia Support	Proposed Sequence in Diagnostic Algorithm
PFO closure	Neuroimaging modality	No	Yes	No	No	High	No *	1
	TCD	Yes	Yes	No	Yes: Shunt grading	Mild	No	2
	TTE	Yes	Yes	No	Yes	Moderate	No	3
	TEE	Yes	Yes	Yes	Yes: Amplitude and length of PFO tunnel	High	Yes	4
	ICE	No	No	Yes	No	High	No	4 §
ASD closure	TTE	Yes	Yes	No	Yes: RV size (RV dilation if RV EDA > 12.6 cm^2^/m^2^ in men, 11.5 cm^2^/m^2^ in women) (17)	Moderate	No	1
	CMR †	Yes	Yes	No	Yes: RV size (RV dilation if RV EDV > 91 mL^2^/m^2^ in men, 80 mL^2^/m^2^ in men) (18)	High	No *	2 †
	TEE	Yes	No ‡	Yes	Yes: ASD border analysis and balloon sizing	High	Yes	3
	ICE	No	No	Yes	No	High	No	4 §

* Selected patients may require conscious sedation; † Routine CMR examination before ASD closure is not required, selected indications include anomalous pulmonary venous return or sinus venosus defect (see text for details); ‡ Pre procedural TEE is considered for patients with marginal anatomical features or unclear diagnosis by TTE, it is discouraged in children due to the need for anesthesia support; § ICE use is limited at this time and it is largely used as an intra-procedural imaging modalities in few centers as a replacement for intra-procedural TEE. ASD = secundum type Atrial Septal Defect; CMR = Cardiac Magnetic Resonance; EDA = End-Diastolic-Area; EDV = End-Diastolic Volume; ICE = IntraCardiac Echocardiography; PFO = Patent Foramen Ovale; RV = Right Ventricle; TCD = Trans-Cranial Doppler; TEE = Trans-Esophageal Echocardiography; TTE = Trans-Thoracic Echocardiography.

**Table 3:** Intra-procedural TEE checklist for PFO and ASD transcatheter closure.

	PFO Closure	ASD Closure
Before vascular access	Free LA appendage Normal aortic and mitral valve No intracardiac mass Atrial septal aneurysm Eustachian valve Chiari Network Accessory fenestration Antero-posterior septal distance	Free LA appendage No significant mitral valve disease Atrial septal aneurysm Eustachian valve Chiari Network Assess border features Multifenestrated ASD Confirm normal pulmonary vein anatomy Bidimensional and color-based shortest and largest ASD diameter 3D-based shortest and largest ASD diameter
After vascular access	Confirm right-to-left shunt at intracardiac bubble study Confirm correct tunnel wiring Confirm wire position in the proper pulmonary vein PFO tunnel amplitude and length	Confirm wire position in the proper pulmonary vein Balloon sizing in stop-flow condition

3D = Three-dimensional; ASD = secundum type Atrial Septal Defect; LA = Left Atrium.

## Data Availability

Not Applicable.

## Supplementary Materials

The following are available online at https://www.mdpi.com/article/10.3390/jcdd8070078/s1, Figure S1 Online Supplementary Material. Multimodality imaging to guide PFO and ASD closure, including pre-closure evaluation and intraprocedural monitoring. * denotes systolic reverse curvature of interventricular septum suggesting for suprasystemic right ventricular pressure at TTE. Single arrow shows presence of large right-to-left shunt at the PFO level. Pre-procedural evaluation for PFO closure includes discarding alternative (rare) source of cardio-embolism such as atrial mixoma, left atrial thrombus (empty arrowhead) or pulmonary AV fistula. Intraprocedural monitoring is devoted to identifying specific anatomical features increasing risk for procedural complication such as large Eustachian valve (double arrow), suboptimal PFO tunnel wiring (filled arrowhead). ASD = secundum type Atrial Septal Defect; AV = artero-venous; PFO = Patent Foramen Ovale; TTE = Trans-Thoracic Echocardiography. Figure S2 Online Supplementary Material. Non-PFO related mechanism of cardio-embolic events. Alternative source of cardioembolic event are presented. AV = artero-venous; LA = Left Atrium; PFO= Patent Forame Ovale. Figure S3 Online Supplementary Material. PFO and accessory fenestration. Left panel shows wrong wire position across an accessory fenestration of septum primum, which allows for right-to-left atrial septal crossing without engaging PFO tunnel. Right panel shows proper tunnel wiring. PFO = Patent Forame Ovale. Figure S4 Online Supplementary Material. Device deployment. (A) Amplatz PFO Occluder (Abbott Laboratories, Abbott Park, IL, USA) sequential deployment. Left panel opening of LA disc, middle panel RA opening with unreleased device, right panel full device release. Pertinent cartoon have been unconditionally provided by Abbott Laboratories, Abbott Park, IL, USA. (B) Sequential deployment of Gore© CardioForm (W. L. Gore & Associates, Inc., Flagstaff, AZ, USA). Right panel shows comparative frame of full device release, please note that the device fabric fluttering is able to generate Doppler signal color-coded by routine TEE monitoring. This feature may be used to better identify disc opening and surrounding structure grasp. Pertinent cartoon have been unconditionally provided by Gore© CardioForm (W. L. Gore & Associates, Inc., Flagstaff, AZ, USA) LA = Left Atrium; PFO= Patent Foramen Ovale; RA = Right Atrium; TEE = Trans-Esophageal Echocardiography. Figure S5 Online Supplementary Material. Complication after PFO closure. Examples of common complications or sub-optimal procedural results after PFO closure. PFO = Patent Foramen Ovale; RA = Right Atrium Figure S6 Online Supplementary Material. Superior sinus venosus defect. TEE feature of superior sinus venosus defect. Please note that in this defect the atrial septum is usually intact and defect is often due to the missing dividing wall between SVC and RUPV. LA = Left Atrium; RA= Right Atrium; RUPV = Right Upper Pulmonary Vein; SVC = Superior Vena Cava; TEE= Trans-Esophageal Echocardiography Figure S7 Online Supplementary Material. Gore© CardioForm ASD Occluder (W. L. Gore & Associates, Inc., Flagstaff, AZ, USA) TEE features. The recently introduced Gore© CardioForm ASD Occluder TEE features are depicted in this composite figure. 3D = Three Dimensional; ASD = secundum type Atrial Septal Defect; LA = Left Atrium; RA = Right Atrium. Figure S8 Online Supplementary Material. Three dimensional TEE and ICE views for PFO and ASD closure. Examples of 3-dimensional TEE views of pre-closure ASD (A,B,C) and PFO (D,E,F,G). H shows ICE view of a multifenestrated ASD. I depicts post-closure three-dimesional TEE view of PFO. Please note the close relationship between device and aortic root in I insert. This anatomic relationship is considered a promoting factor for device erosion into the aortic root ASD = secundum type Atrial Septal Defect; ICE = IntraCardiac Echocardiography; PFO = Patent Foramen Ovale; TEE = TransEsophageal Echocardiography. Figure S9 Online Supplementary Material. Sizing during ASD closure. Comparing TEE (left) and fluoroscopic (right) view during balloon sizing of ASD. ASD = secundum type Atrial Septal Defect; TEE = Trans-Esophageal Echocardiography. Figure S10 Online Supplementary Material. Right upper pulmonary vein border. High esophageal bicaval TEE view with posterior probe rotation to better delineate RUPV ASD border and atrial roof. ASD = secundum type Atrial Septal Defect; RUPV = Right Upper Pulmonary Vein. Online Supplementary Material Video Caption. Video S1 Online Supplementary Material. Atrial septal aneurysm. TEE views of multiple examples of redundant septal aneurysm with 3D reconstruction. Please note the heterogenity of morphological spectrum of atrial septal anatomy. TEE = Trans-Esophageal Echocardiography. Video S2 Online Supplementary Material. TCD contrast study. Our protocol for TCD contrast study includes forming the contrast media using 9 mL of saline, 1 mL of air and 1 mL of patient blood. Proper patient coaching regarding Valsalva maneuver and appropriate timing of media infusion are key details to increase test sensitivity. TCD = Trans-Cranial Doppler Video S3 Online Supplementary Material. Right-to-left shunt at PFO level TEE long axis view showing intraprocedural injection of contrast media from the femoral vein (please note that the contrast is traveling from the inferior vena cava, compared to ambulatory TCD studies where contrast media reaches the right atrium usually rom the superior vena cava). There is severe right-to-left shunt indicated by left atrial passage of contrast media through the PFO within three cardiac cycles from complete right atrial opacification. TCD = Trans-Cranial Doppler; TEE = Trans-Eshophageal Echocardiography; PFO = Patent Foramen Ovale. Video S4 Online Supplementary Material. Pulmonary artero-venous fistula. Composite video showing baseline positive TCD in a patient with PFO and pulmonary artero-venous fistula. In the second and third clip, there is severe contrast media coming back to the left atrium from the pulmonary vein after PFO closure (please note the device located at the level of PFO). Pertinent selective pulmonary angiography (fourth and fifth clip) confirmes the presence of a relatively larger pulmonary artero-venous fistula of the left lung. Final angiography shows complete fistula occlusion after plug-embolization. PFO = Patent Forame Ovale; TCD = Trans-Cranial Doppler. Video S5 Online Supplementary Material. Device release. Subtle device position shifting at the time of final release. Video S6 Online Supplementary Material. Device traction test, loosing SVC border. TEE modified short axis view (60°) showing pre-release device traction. In this case this maneuver is associated with device dislocation due to the loss of SVC border by left atrial disc. SVC = Superior Vena Cava. Video S7 Online Supplementary Material. Device traction test, thorus aorticus residual shunt. TEE short axis view (45°) shows pre-release device traction. In this particular case this maneuver is associated with occurrence of residual (and transitory) shunt between the device and thorus aorticus. This needs to be distinguished from intra-device shunt and it may be associated with increased propensity to device embolization. TEE = Trans-Eshophageal Echocardiography Video S8 Online Supplementary Material. Device embolization into the LV. Low esophageal four-chamber TEE view (20°) shows embolized device into left ventricular cavity. Emergent surgical intervention was required to remove the device and perform atrial septal defect closure. TEE = Trans-Eshophageal Echocardiography.

## Abbreviations

AO	AOrta
AoV	Aortic Valve
ASD	Atrial Septal Defect
AV	Artero-Venous
AVV	Atrio-Ventricular Valves
CMR	Cardiac Magnetic Resonance
DWI	Diffusion-Weighted magnetic resonance Imaging
CS	Coronary Sinus
EDV	End-Diastolic Volume
ESUS	Embolic Stroke of Unknown Source
ESV	End-systolic Volume
FO	Fossa Ovalis
HITS	High Intensity Transient Signals
ICE	Intracardiac Echocardiography
IVC	Inferior Vena Cava
LA	Left Atrium
PFO	Patent Foramen Ovale
RA	Right Atrium
PV	Pulmonary Valve
RV	Right Ventricle
SVC	Superior Vena Cava
TCD	Trans-Cranial Doppler
TEE	Trans-Esophageal Echocardiography
TTE	Trans-Thoracic Echocardiography
TV	Tricuspid Valve
