# Supplementary material for: Transcatheter Closure of PFO and ASD: Multimodality Imaging for Patient Selection and Perioperative Guidance
Source: J Cardiovasc Dev Dis. 2021 Jul 3;8(7):78. doi: 10.3390/jcdd8070078 (PMC8306204; doi:10.3390/jcdd8070078)
Supplement: Supplementary file 1 [file jcdd-08-00078-s001.zip › jcdd-1269861-sup.pdf]

Online Supplementary Material

Appendix A.

Focused anatomy of atrial septum

There is wide anatomical heterogeneity in the anatomy of atrial septum. Consistent finding of a normally formed atrial septum include the presence of a relatively centered area of thinner tissue that is formally defined as the fossa ovalis (Figure S1). This portion of the septum is formed by the embryologic remnant of septum primum. During embryonic life the septum primum forms primarily from the embryonic venous valve starting the septation process of the primitive atrium. Subsequently, the atrial wall starts to fold cranially, this folding process does create a thicker septum secundum located to the right side of the septum primum (Figure S2). Septation process includes post-natal fusion of the two septa allowing for complete separation of pulmonary and systemic venous return. Incomplete fusion of these two septa is frequent in humans and represent the embryologic substrate of PFO (Figure S2). Pathology study reveals PFO prevalence approximately in 25% of study population. In vivo-study performed in patients with clinically evident cerebral or systemic ischemic event showed an interesting fluctuation of such prevalence with higher PFO prevalence in low cardiovascular risk population, suggesting a causal relationship between PFO and systemic cardioembolic event through paradoxical embolization. The width and length of the PFO tunnel can be widely different from case to case, depending on the degree of separation and overlapping of the two septa (Figure S2). The septum primum can be redundant and mobile forming an atrial septal aneurysm and it can harbour one or more fenestrations creating additional communications between the atria. The septum secundum can vary in thickness, depending on the amount of fat tissue, up to more than 10 mm (lipomatous hypertrophy). Therefore, the anatomical features of the PFO are consistently variable and additional anatomical structures such as a redundant Eustachian valve or a Chiari network, can further add to the complexity of its anatomy.

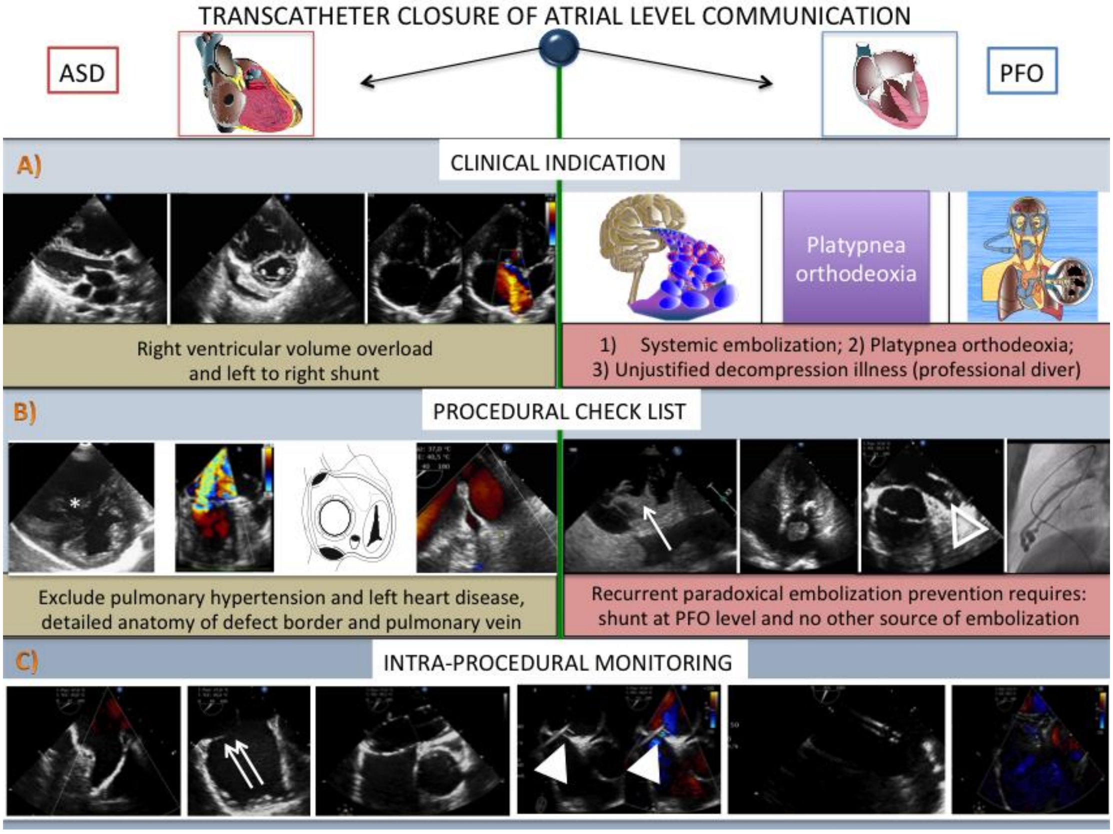

Online Supplementary Material Figure Caption

Figure S1 Online Supplementary Material.

Multimodality imaging to guide PFO and ASD closure, including pre-closure evaluation and intraprocedural monitoring. * denotes systolic reverse curvature of interventricular septum suggesting for suprasystemic right ventricular pressure at TTE. Single arrow shows presence of large right-to-left shunt at the PFO level. Pre-procedural evaluation for PFO closure includes discarding alternative (rare) source of cardio-embolism such as atrial mixoma, left atrial thrombus (empty arrowhead) or pulmonary AV fistula. Intraprocedural monitoring is devoted to identifying specific anatomical features increasing risk for procedural complication such as large Eustachian valve (double arrow), suboptimal PFO tunnel wiring (filled arrowhead). ASD= secundum type Atrial Septal Defect; AV= artero-venous; PFO= Patent Foramen Ovale; TTE= Trans-Thoracic Echocardiography.

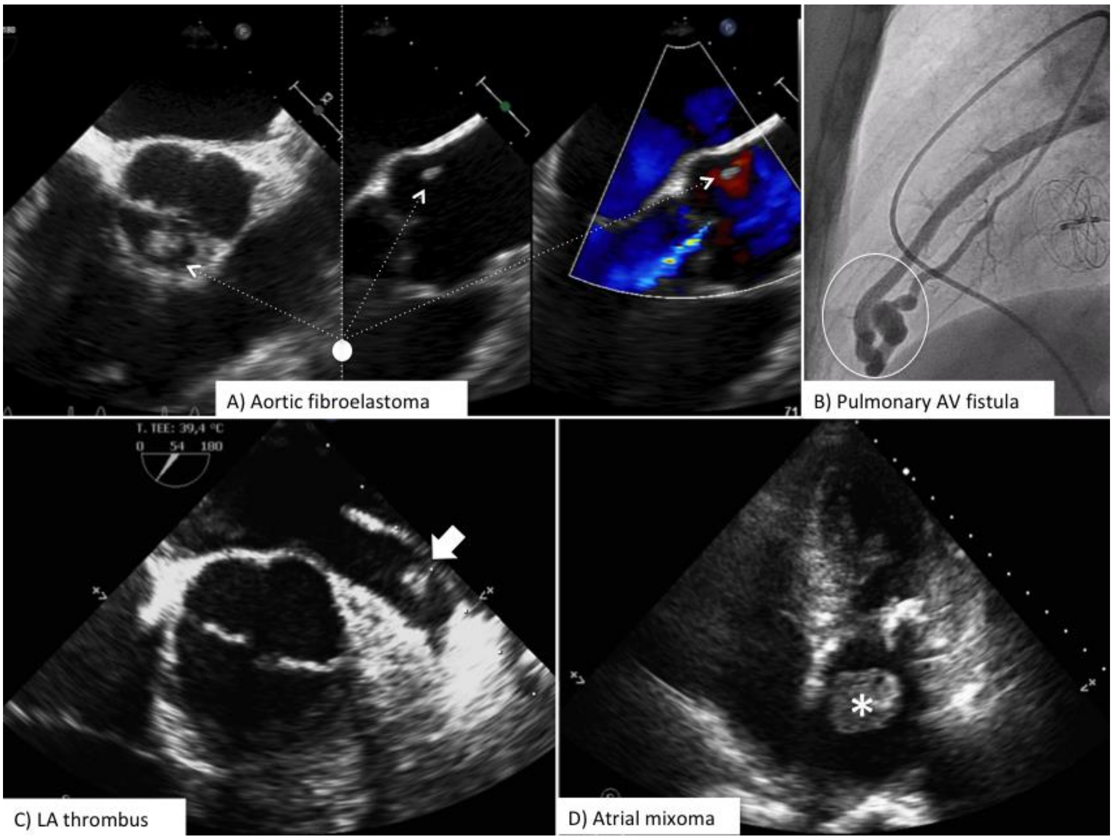

Figure S2 Online Supplementary Material. Non-PFO related mechanism of cardio-embolic events.
Alternative source of cardioembolic event are presented.
AV= artero-venous; LA= Left Atrium; PFO= Patent Forame Ovale.

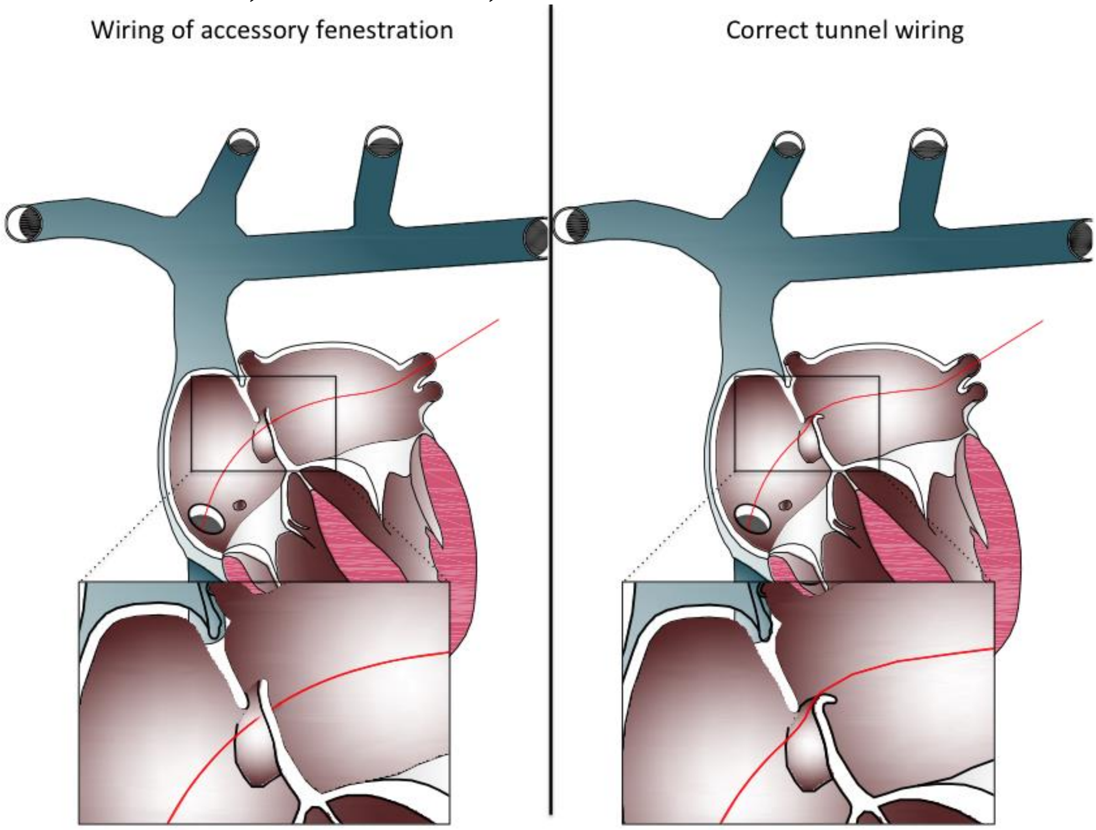

Figure S3 Online Supplementary Material. PFO and accessory fenestration.

Left panel shows wrong wire position across an accessory fenestration of septum primum, which allows for right-to-left atrial septal crossing without engaging PFO tunnel. Right panel shows proper tunnel wiring.
PFO= Patent Forame Ovale.

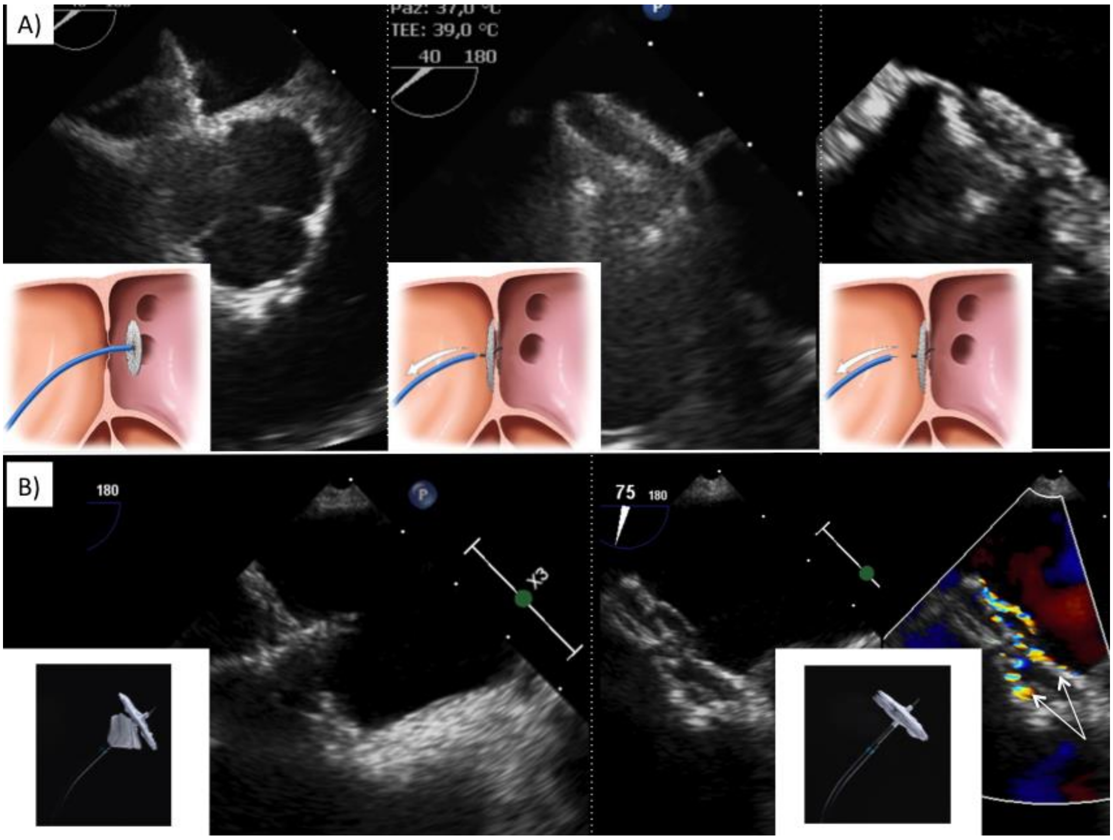

Figure S4 Online Supplementary Material. Device deployment.
A) Amplatz PFO Occluder (Abbott Laboratories, Abbott Park, Illinois, USA) sequential deployment. Left panel opening of LA disc, middle panel RA opening with unreleased device, right panel full device release. Pertinent cartoon have been unconditionally provided by Abbott Laboratories, Abbott Park, Illinois, USA.
B) Sequential deployment of Gore© CardioForm (W. L. Gore & Associates, Inc. Flagstaff, Arizona, USA). Right panel shows comparative frame of full device release, please note that the device fabric fluttering is able to generate Doppler signal color-coded by routine TEE monitoring. This feature may be used to better identify disc opening and surrounding structure grasp.
Pertinent cartoon have been unconditionally provided by Gore© CardioForm (W. L. Gore & Associates, Inc. Flagstaff, Arizona, USA)
LA= Left Atrium; PFO= Patent Foramen Ovale; RA= Right Atrium; TEE= Trans-Esophageal Echocardiography.

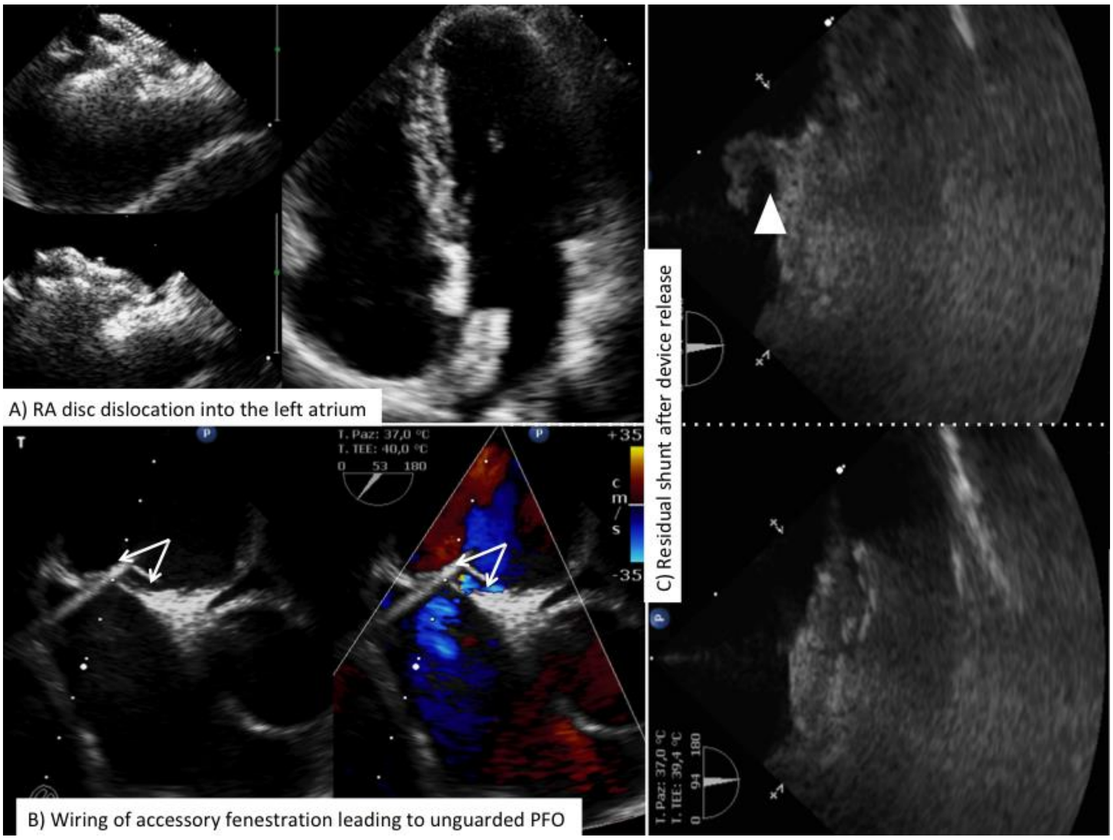

Figure S5 Online Supplementary Material. Complication after PFO closure. Examples of common complications or sub-optimal procedural results after PFO closure.
PFO= Patent Foramen Ovale; RA= Right Atrium

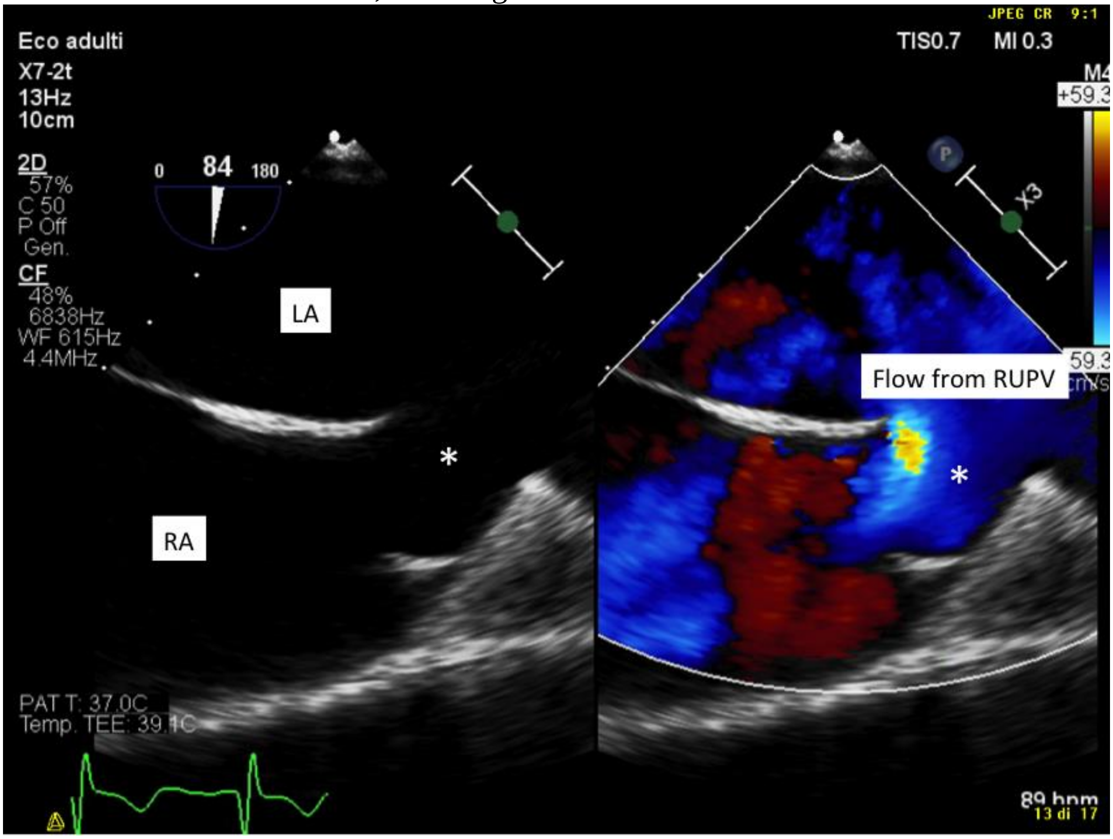

Figure S6 Online Supplementary Material. Superior sinus venosus defect.

TEE feature of superior sinus venosus defect. Please note that in this defect the atrial septum is usually intact and defect is often due to the missing dividing wall between SVC and RUPV.
LA= Left Atrium; RA= Right Atrium; RUPV= Right Upper Pulmonary Vein; SVC= Superior Vena Cava; TEE= Trans-Esophageal Echocardiography

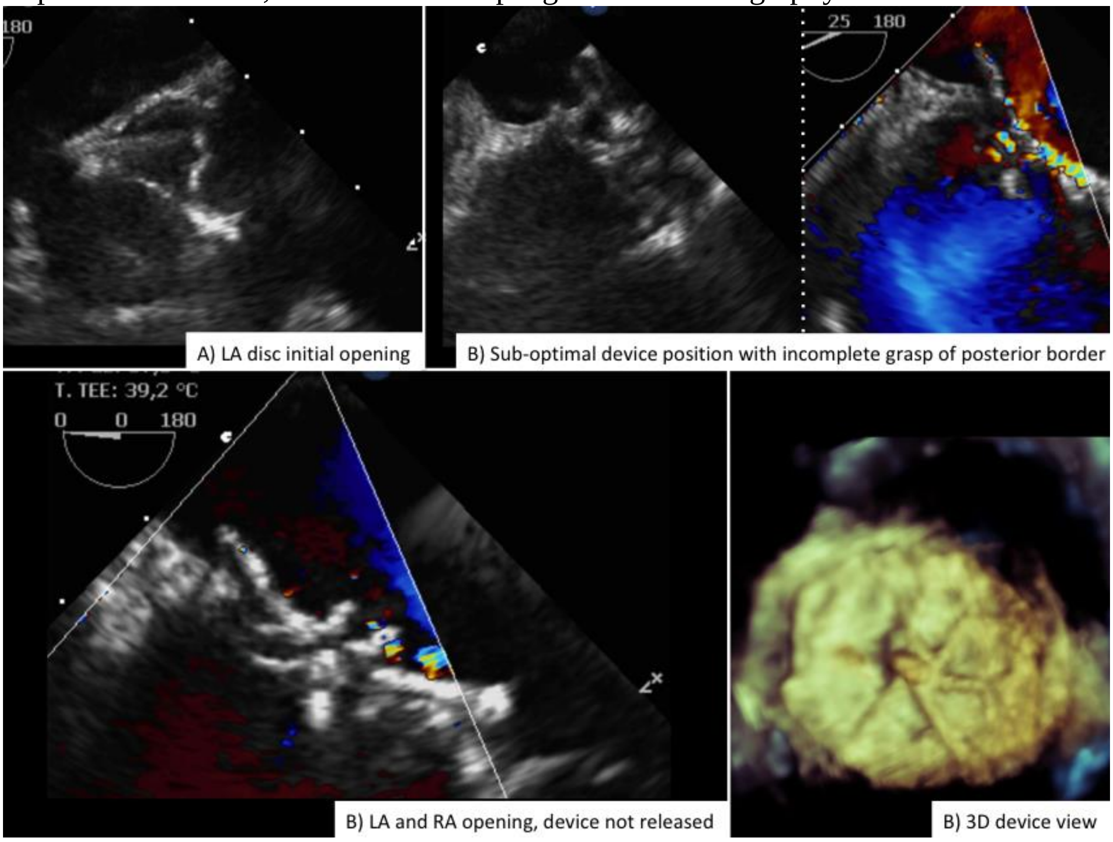

Figure S7 Online Supplementary Material. Gore© CardioForm ASD Occluder (W. L. Gore & Associates, Inc. Flagstaff, Arizona, USA) TEE features.
The recently introduced Gore© CardioForm ASD Occluder TEE features are depicted in this composite figure.
3D= Three Dimensional; ASD= secundum type Atrial Septal Defect; LA= Left Atrium; RA= Right Atrium.

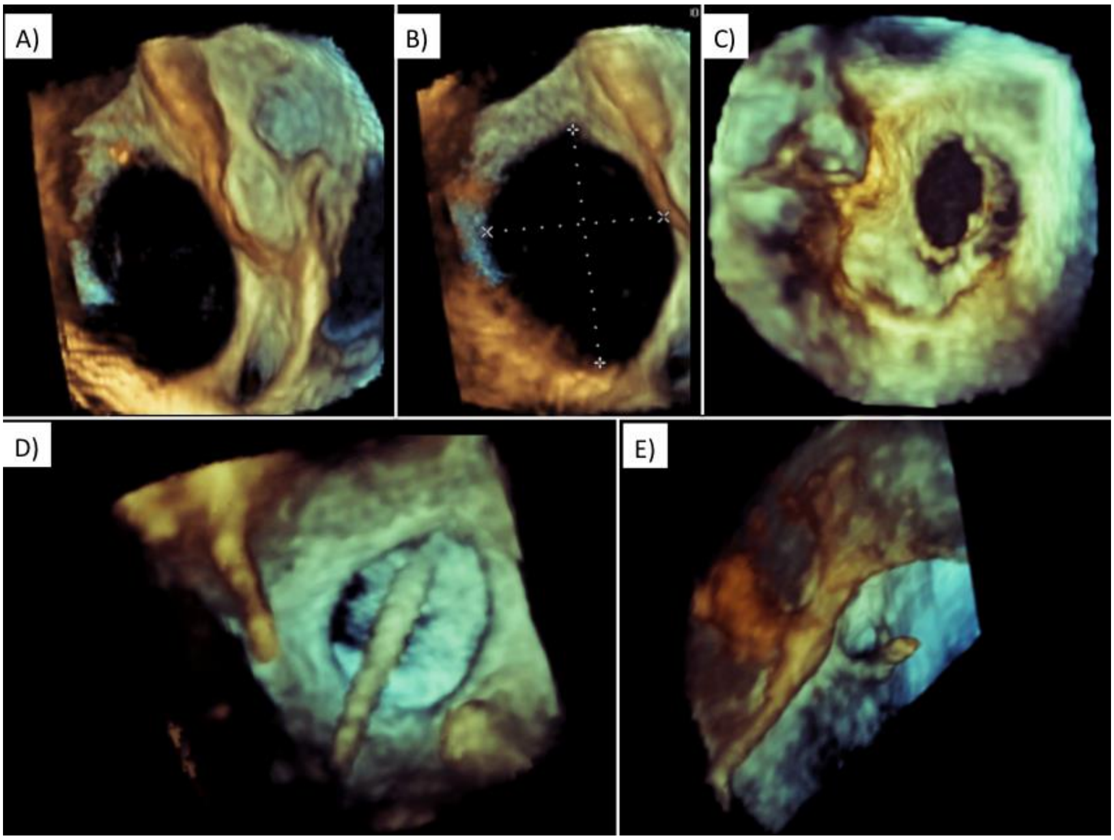

(**a**)

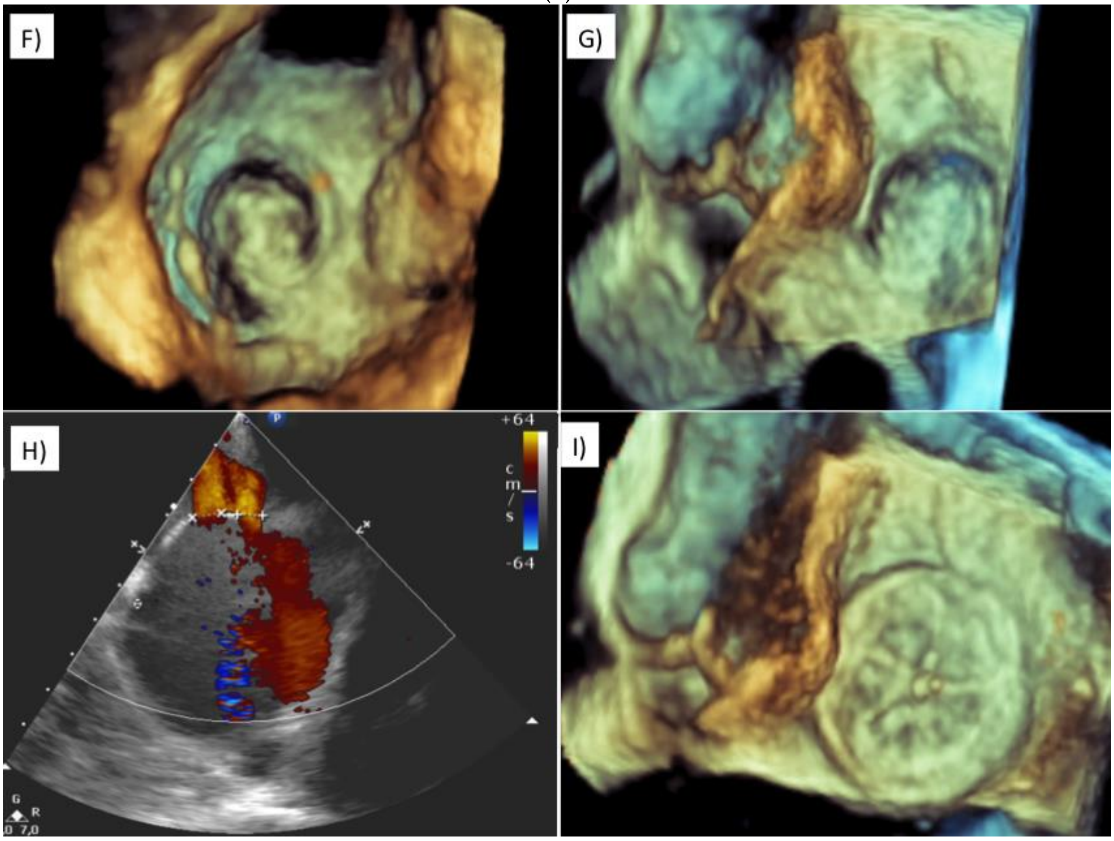

(**b**)

Figure S8 Online Supplementary Material. Three dimensional TEE and ICE views for PFO and ASD closure.

Examples of 3-dimensional TEE views of pre-closure ASD (A,B,C) and PFO (D,E,F,G). H shows ICE view of a multifenestrated ASD. I depicts post-closure three-dimesional TEE view of PFO. Please note the close relationship between device and aortic root in I insert. This anatomic relationship is considered a promoting factor for device erosion into the aortic root
ASD= secundum type Atrial Septal Defect; ICE= IntraCardiac Echocardiography; PFO= Patent Foramen Ovale; TEE= TransEsophageal Echocardiography

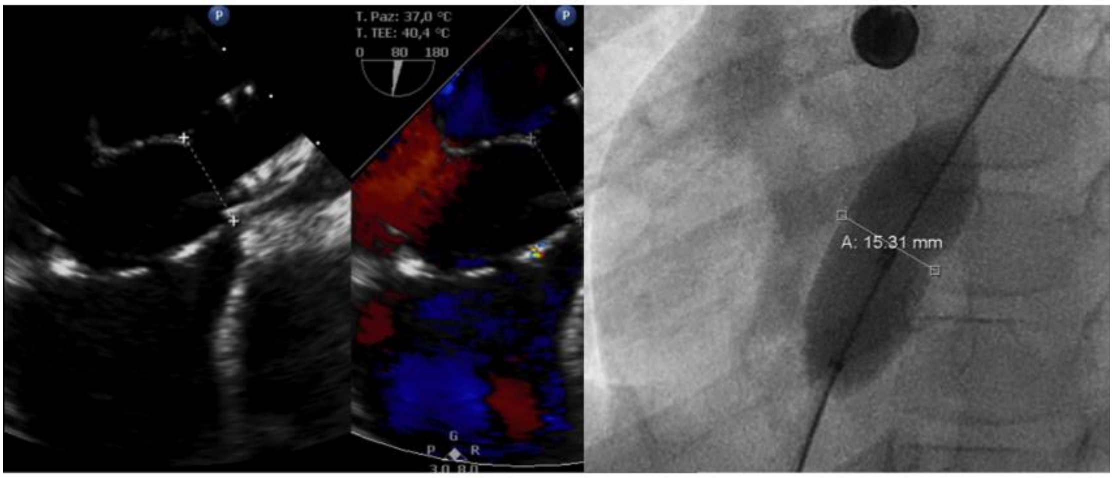

Figure S9 Online Supplementary Material. Sizing during ASD closure
Comparing TEE (left) and fluoroscopic (right) view during balloon sizing of ASD. ASD= secundum type Atrial Septal Defect; TEE= Trans-Esophageal Echocardiography.

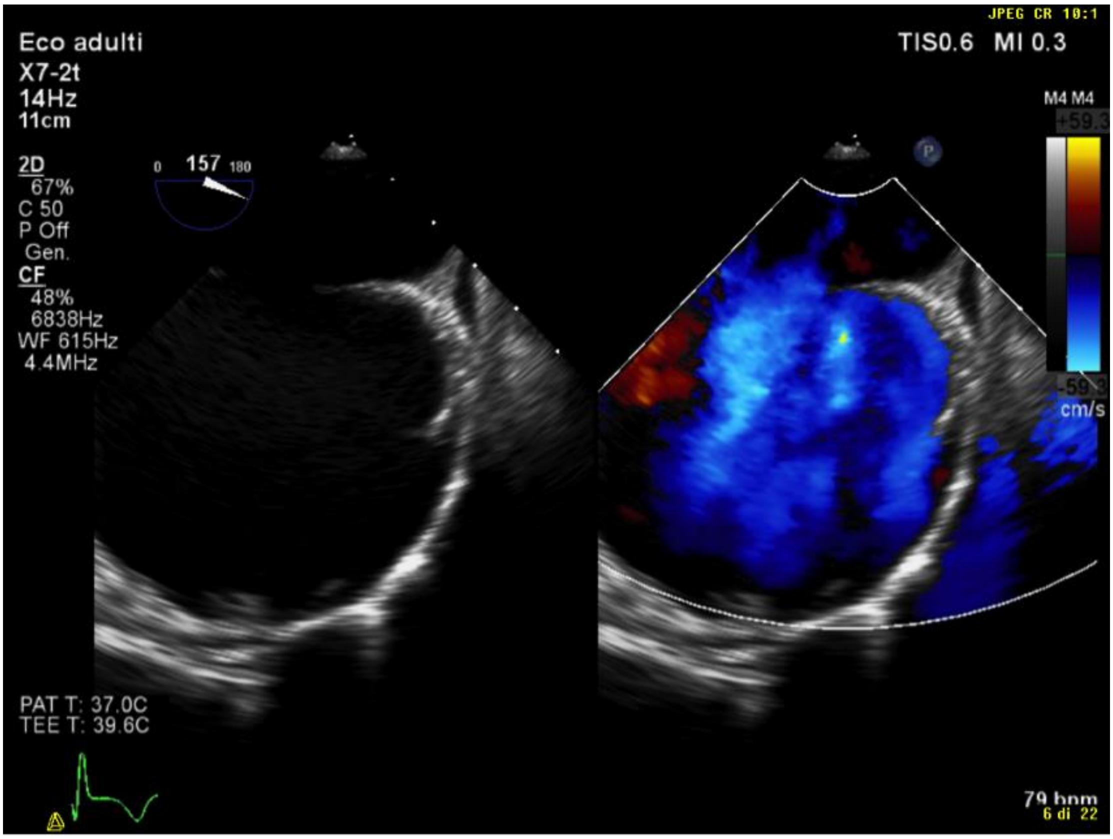

Figure S10 Online Supplementary Material. Right upper pulmonary vein border
High esophageal bicaval TEE view with posterior probe rotation to better delineate RUPV ASD border and atrial roof.
ASD= secundum type Atrial Septal Defect; RUPV= Right Upper Pulmonary Vein.

Online Supplementary Material Video Caption.

Video S1 Online Supplementary Material. Atrial septal aneurysm.
TEE views of multiple examples of redundant septal aneurysm with 3D reconstruction. Please note the heterogenity of morphological spectrum of atrial septal anatomy.
TEE= Trans-Esophageal Echocardiography.

Video S2 Online Supplementary Material. TCD contrast study.
Our protocol for TCD contrast study includes forming the contrast media using 9ml of saline, 1ml of air and 1ml of patient blood. Proper patient coaching regarding Valsalva maneuver and appropriate timing of media infusion are key details to increase test sensitivity.
TCD= Trans-Cranial Doppler

Video S3 Online Supplementary Material. Right-to-left shunt at PFO level
TEE long axis view showing intraprocedural injection of contrast media from the femoral vein (please note that the contrast is traveling from the inferior vena cava, compared to ambulatory TCD studies where contrast media reaches the right atrium usually rom the superior vena cava). There is severe right-to-left shunt indicated by left atrial passage of contrast media through the PFO within three cardiac cycles from complete right atrial opacification.

TCD= Trans-Cranial Doppler; TEE= Trans-Eshophageal Echocardiography; PFO= Patent Foramen Ovale.

Video S4 Online Supplementary Material. Pulmonary artero-venous fistula.
Composite video showing baseline positive TCD in a patient with PFO and pulmonary artero-venous fistula. In the second and third clip, there is severe contrast media coming back to the left atrium from the pulmonary vein after PFO closure (please note the device located at the level of PFO). Pertinent selective pulmonary angiography (fourth and fifth clip) confirmes the presence of a relatively larger pulmonary artero-venous fistula of the left lung. Final angiography shows complete fistula occlusion after plug-embolization.
PFO= Patent Forame Ovale; TCD= Trans-Cranial Doppler.

Video S5 Online Supplementary Material. Device release.
Subtle device position shifting at the time of final release.
Video S6 Online Supplementary Material. Device traction test, loosing SVC border.
TEE modified short axis view (60 °) showing pre-release device traction. In this case this maneuver is associated with device dislocation due to the loss of SVC border by left atrial disc.
SVC= Superior Vena Cava.
Video S7 Online Supplementary Material. Device traction test, thorus aorticus residual shunt.
TEE short axis view (45 °) shows pre-release device traction. In this particular case this maneuver is associated with occurrence of residual (and transitory) shunt between the device and thorus aorticus. This needs to be distinguished from intra-device shunt and it may be associated with increased propensity to device embolization.
TEE= Trans-Eshophageal Echocardiography
Video S8 Online Supplementary Material. Device embolization into the LV.
Low esophageal four-chamber TEE view (20 °) shows embolized device into left ventricular cavity. Emergent surgical intervention was required to remove the device and perform atrial septal defect closure.
TEE= Trans-Eshophageal Echocardiography
